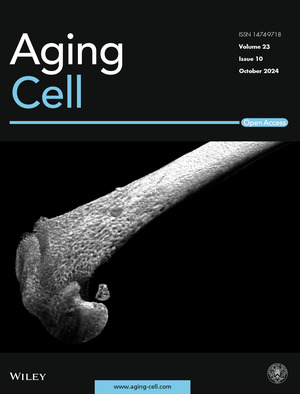# Additional Cover

**DOI:** 10.1111/acel.14381

**Published:** 2024-10-09

**Authors:** Yuichiro Ukon, Takashi Kaito, Hiromasa Hirai, Takayuki Kitahara, Masayuki Bun, Joe Kodama, Daisuke Tateiwa, Shinichi Nakagawa, Masato Ikuta, Takuya Furuichi, Yuya Kanie, Takahito Fujimori, Shota Takenaka, Tadashi Yamamuro, Satoru Otsuru, Seiji Okada, Masakatsu Yamashita, Takeshi Imamura

## Abstract

Cover legend: The cover image is based on the Article *Cellular senescence by loss of Men1 in osteoblasts is critical for age‐related osteoporosis* by Yuichiro Ukon et al., https://doi.org/10.1111/acel.14254